# Feasibility of a Progesterone-Modified Natural Protocol for Frozen Embryo Transfer: Protocol for a Pilot Cohort Study

**DOI:** 10.2196/66579

**Published:** 2025-04-11

**Authors:** Alexandra Churchill, Ektoras Georgiou, Veronica Abruzzo, Alex Polyakov, Wan Tinn Teh

**Affiliations:** 1 Reproductive Services Unit The Royal Women's Hospital Melbourne Australia; 2 Melbourne Medical School The University of Melbourne Melbourne Australia; 3 The Department of Obstetrics & Gynaecology The University of Melbourne Melbourne Australia; 4 Melbourne IVF (East Melbourne) Melbourne Australia; 5 Genea Fertility (East Melbourne) Melbourne Australia; 6 City Fertility (East Melbourne) Melbourne Australia

**Keywords:** frozen embryo transfer, fertility care, reproductive health, infertility, progesterone-modified natural cycle protocol, in vitro fertilization

## Abstract

**Background:**

With the existence of various frozen embryo transfer (FET) methods currently used in the field of assisted reproductive technologies, the debate surrounding which of these is superior remains. All FET protocols aim to prime the endometrium and time embryo transfer during the window of implantation. Current methods include the true natural cycle FET (tNFET), modified natural cycle FET, artificial cycle FET, and ovulation induction. Each of these harbors, distinct advantages and disadvantages, namely, surrounding the timing of transfer and flexibility conferred through this process. More recently, a newer approach has been used whereby the need to monitor or trigger ovulation is circumvented, with luteal phase support commenced once a certain follicle diameter and endometrial thickness criteria are met but before ovulation. However, the research into this protocol has certain important limitations that our study seeks to address.

**Objective:**

This study aims to assess the feasibility of a progesterone-modified natural cycle protocol for FET. The primary outcome will be the presence of a corpus luteum on ultrasound scans on the day of embryo transfer. The secondary outcomes will include the number of clinic visits required per patient undergoing the protocol, biochemical pregnancy rate, and clinical pregnancy rate.

**Methods:**

We will conduct a prospective cohort study, recruiting 20 women undertaking FET at the Public Fertility Care of The Royal Women’s Hospital in Melbourne, Australia. These women will be matched to a control group who have undergone the tNFET protocol within the preceding 12 months of the study start date.

**Results:**

This project received ethics approval on July 17, 2024, with commencement of the study in September 2024, aiming for a duration of completion of 9 months. The completion of the follow-up and submission of the study for publication are anticipated for September 2025.

**Conclusions:**

After this preliminary study, the aim would be to progress to a noninferiority randomized controlled trial to compare the progesterone-modified natural cycle protocol for FET to the tNFET.

**International Registered Report Identifier (IRRID):**

PRR1-10.2196/66579

## Introduction

Frozen embryo transfer (FET) has been increasingly used over recent decades in Europe [1,2] and Australia [2]. With advancements in cryopreservation techniques and increasing use of FET, debate still exists around the optimal method of endometrial preparation to achieve the best possible pregnancy rates and improve various maternal and neonatal outcomes. The most widely used protocols for endometrial preparation include the natural cycle FET (NFET), artificial cycle FET (AFET), and ovulation induction methods [3], with the latter two protocols usually reserved for anovulatory women. While the true NFET (tNFET) relies on monitoring for a surge of endogenous luteinizing hormone (LH) and rise in progesterone (P4) as markers of ovulation to time P4 supplementation start and embryo transfer, the AFET protocol involves exogenous hormone (estrogen, followed by P4) administration to mimic the natural cycle and prime the endometrium. Furthermore, while the AFET confers greater flexibility in terms of timing FET precisely, there is evidence to suggest lower overall implantation rates and higher miscarriage rates from this technique [4]. The literature also suggests there may be a higher rate of obstetric complications for both mother and fetus, including pre-eclampsia [5] and fetal macrosomia [6]. Therefore, the advantage of tNFET centers on avoiding excessive exogenous hormone administration and the resulting adverse events (AEs) linked to AFET. However, the inflexibility conferred by the tNFET in terms of the need to closely monitor endometrial thickness, follicle size, and hormone levels may require multiple blood tests and 7-day in-vitro fertilization clinic availability. Further, the ovulation induction protocol involves the expense and invasiveness of using medication without removing the need for a 7-day service or the need for blood tests. Therefore, a protocol that achieves ovulation and gives a degree of flexibility to the timing of embryo transfer would be highly appealing to both patients and in-vitro fertilization clinics.

To address these challenges, some clinics use the modified NFET (mNFET) protocol where exogenous human chorionic gonadotropin (hCG) is administered to trigger ovulation when a dominant follicle of typically 17 mm or more is detected, thereby conferring some flexibility in scheduling FET while still relying on the woman’s natural cycle [7]. Retrospective cohort studies have shown favorable outcomes in both the tNFET and mNFET as compared to the AFET [8]. Notably, two recent randomized controlled trials (RCTs) showed similar rates of pregnancy in the mNFET as compared to the tNFET, with higher implantation rates in the mNFET cohort [7,9]. However, at least one study has reported better outcomes with tNFET [10], and the most recent Cochrane systematic review on cycle regimens of FET did not compare tNFET and mNFET [3].

To further explore the potential flexibility conferred by the mNFET method while mitigating the lack of flexibility in scheduling the transfer, a prospective case series proof-of-concept study by Weiss et al [11] has trialed a novel approach whereby timing the FET to the endogenous LH surge is circumvented. Instead, P4 luteal phase support (LPS) via vaginal pessary is commenced once a mature follicle of >12 mm is identified in ultrasound scans and the lining of the endometrium is sufficiently thick at >7 mm, with FET scheduled 2-5 days from this point, depending on the stage of the embryo at the time of cryopreservation. It appears that this P4-modified natural protocol for FET (P4mNFET) provides a simultaneous advantage of both retaining a natural cycle, with authors suggesting ovulation took place regardless, as well as conferring greater flexibility for the scheduling of embryo transfer without compromising clinical pregnancy rates. It should be noted that the authors report a degree of variation in the timing of P4 initiation in relation to the last ultrasound scan without any further blood tests. Importantly, no AEs were reported from this study of 42 participants. A more recent retrospective cohort study comparing this method to AFET demonstrated comparable outcomes in terms of clinical pregnancy, miscarriage, and live birth rates between the methods [12]. A further single-center retrospective cohort study reported similar results when comparing outcomes from patients undergoing FET cycles within the natural and artificial protocols, as compared to a P4mNFET protocol [13]. Taken together, P4mNFET may confer greater flexibility to clinicians and patients in timing FET, negating the requirement to await ovulation, with comparable outcomes.

Overall, this study aims to provide greater flexibility in timing transfer by providing participants with vaginal pessaries of P4 to be used before FET and, subsequently, confirming the absence of elevated endogenous P4 on the day of P4 supplementation start and confirming the presence of a corpus luteum on an ultrasound scan. It is hypothesized that the potential advantages of this P4mNFET protocol, over triggering and manipulating ovulation, may include the increased flexibility in the timing of commencing P4 without the requirement of a large follicle, the elimination of the importance of pinpointing the exact timing of ovulation, and the potential cost-saving to the patient and clinic as a result of these components. It is anticipated that the outcomes of this pilot cohort study may inform the planning and development of a subsequent noninferiority RCT to further reinforce the utility of such a protocol.

## Methods

### Study Type

The proposed study will adopt a pilot cohort methodology consisting of 12 weeks of intervention and follow-up monitoring of participants for primary and secondary outcomes. Secondary outcomes will be compared with retrospective data from the tNFET control group.

### Participants

The study population will consist of 20 women undertaking FET cycles at the Public Fertility Care of The Royal Women’s Hospital (RWH), Melbourne, Australia. The recruitment process will commence at this location, whereby clinicians and nursing teams will be invited to screen the patients they interact with for eligibility for the study. These staff members will be trained on appropriate screening according to the study protocol and will be delegated by the principal investigator. Eligible patients will then be approached for study by team members who are not directly involved in potential participants’ clinical care and, therefore, will not have had any clinical interactions with the participant. All patients will be provided with a patient informed consent form (Multimedia Appendix 1) to ensure they are familiar with the protocol requirements and understand their rights as participants. Additionally, they will be provided with a patient information document created by the research team (Multimedia Appendix 2) to assist with patient education surrounding the use of the P4 pessary during the study.

Inclusion criteria consist of women with regular cycles defined as 21-35 days, women younger than 40 years at the start of a cycle, and women whose BMI ranges from 18 to 35, inclusively.

The exclusion criteria include anovulatory women, women who are 40 years or older at the start of the cycle, and women who have preexisting contraindications to exogenous P4 supplementation, such as those with liver disease or thromboembolic disease. The exclusion criteria will also include the use of additional LPS (eg, subcutaneous), women with uterine pathology, including congenital malformations of the female reproductive tract, endometrial polyps, intrauterine adhesions, adenomyosis, and leiomyoma. Regarding the retrospective element of the study, patients for whom inclusion criteria information is lacking will not be included in the study.

The control group will consist of retrospective data from 20 women, matched against inclusion and exclusion criteria, within the Public Fertility Services of the RWH data bank. These controls would have undergone tNFET at the service provider within the preceding 12 months of the study start date.

All participant data will be stored securely in the database of the RWH Reproductive Services Unit (RSU), ensuring the security of patients’ confidential information. Furthermore, on completion of the study, all patient information will be securely stored within the hospital server with restricted access for a minimum of 15 years, with custodial responsibilities given to the principal investigator.

### Outcome Measures

The primary outcome will be the presence of corpus luteum with a characteristic “ring of fire” appearance on the transabdominal ultrasound on the day of embryo transfer.

The secondary outcomes consist of the number of clinic visits required per patient undergoing this protocol, the biochemical pregnancy rate defined as the detection of βhCG in serum or urine [14], and the clinical pregnancy rate defined as the ultrasonographic visualization of one or more gestational sacs [14].

### Randomization

This is a pilot prospective cohort study without randomization.

### Intervention: P4mNFET Protocol

**Figure 1:**
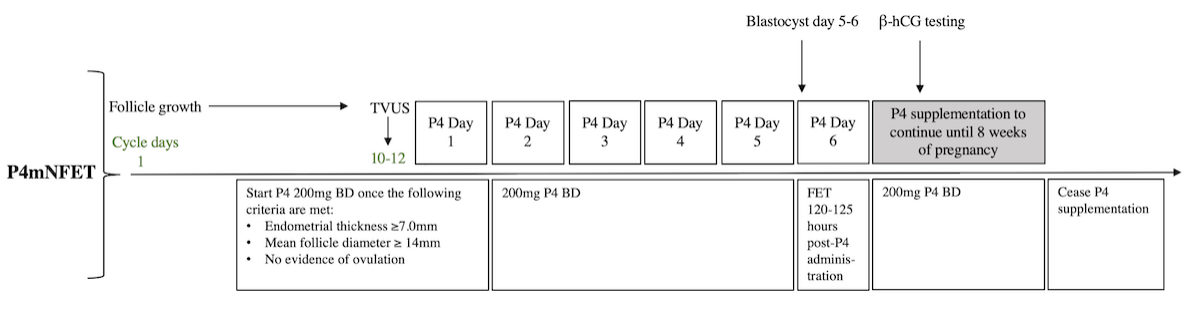
Visual representation of the patient journey with P4mNFET protocol. FET: frozen embryo transfer; hCG: human chorionic gonadotropin; P4: progesterone; P4mNFET: progesterone-modified natural cycle protocol for frozen embryo transfer; TVUS: transvaginal ultrasound.

Participants will undergo active treatment for 2-8 weeks, with a follow-up period of up to 12 weeks or gestation. For a patient with a 28-day menstrual cycle, on days 10-12 (Figure 1), a transvaginal ultrasound will be performed to evaluate for an appropriate endometrial thickness of 7.0 mm and a mean follicle diameter of 14.0 mm. If these ultrasound criteria are met, the patient will undergo blood tests for estrogen, LH, and P4 on the same day; should levels of P4 be under 5 nmol/L, exogenous P4 supplementation via vaginal pessary will then commence on the same day, with FET scheduled 120-125 hours post P4 administration. P4 pessary, ORIPRO (Orion Laboratories Pty Ltd T/A Perrigo Australia, Balcatta, Western Australia), at a dose of 200 mg twice daily, will be the only form of LPS for patients undergoing P4mNFET. Following FET, serum hCG will be taken 10 days later. If positive, LPS via P4 pessary at 200 mg twice daily will continue to 8 weeks’ gestation with a pregnancy scan between 6-7 weeks. If negative, the patient will be advised to cease LPS. In the instances where P4 levels are greater than 5 nmol/L, the patient will be removed from the study.

### Control: tNFET Protocol

The control group will consist of patients, matched to the inclusion and exclusion criteria, who would have undergone tNFET at the RWH RSU. Patients undertake an ultrasound on days 10-12 of their cycle for those with a 28-day cycle. Upon detection of a dominant follicle of 18 mm or more and an endometrial thickness of 7 mm or more, serum LH and P4 are taken. An LH surge is defined as >25 IU/L in the context of P4 <5 nmol/L. Ovulation is defined as a P4 >5 nmol/L. LPS, in the form of a vaginal ORIPRO 200 mg pessary (Orion Laboratories Pty Ltd T/A Perrigo, Australia), is commenced on day 1 post ovulation, and embryo transfer is carried out 6 days post LH surge.

### Statistical Analysis and Sample Size

Given the qualitative and binary nature of the primary outcome, no statistical testing is proposed for this. Secondary outcomes will undergo *t* tests and Mann-Whitney *U* tests for data that are normally and not normally distributed, respectively. Should there be significant variation in the key baseline variables between the study and the control group, a multivariate analysis will be performed.

### Safety Monitoring and Reporting

Safety oversight for the study will be carried out under the direction of the independent safety monitor working within the framework of the Data and Safety Monitoring Board Charter to ensure an objective assessment of the safety and efficacy of the study. AEs deemed secondary to the administration of the pessary will be monitored and reported accordingly by either the investigators or the independent safety monitor, from administration to the end of follow-up. Relevant physical examinations or investigations will then be carried out to ensure the effects of the AE are managed accordingly. The primary investigator will record the AE appropriately into the patient’s medical record or study shadow file.

### Integration and Dissemination of Findings

On completion of the study, primary and secondary outcomes will be disseminated to investigators, ensuring the confidentiality of patient information. Findings will be synthesized and communicated to colleagues of the RWH RSU and Human Research Ethics Committee, as well as in further peer-reviewed publications related to the RCT that may be conducted following the completion of this cohort study. Additionally, findings will be communicated confidentially in potential presentations at national and international conferences.

## Results

This project was conceived in November 2023. It was subsequently approved by the RWH Human Research Ethics Committee on July 17, 2024. Local governance approval was granted on August 28, 2024.

Commencement of the study began in September 2024, aiming for a duration of completion of 9 months, and completion of follow-up and submission of the study for publication in September 2025.

## Discussion

### Overview

We have designed a pilot cohort nonrandomized study to assess the feasibility of a novel P4mNFET protocol that circumvents the need to monitor for an endogenous LH surge to pinpoint ovulation to schedule FET. This protocol, therefore, may combine the advantages of the tNFET, AFET, and mNFET into one protocol that provides greater flexibility for the timing of transfer with fewer requirements for surveillance and no impact on pregnancy outcomes.

### Anticipated Challenges

As with any study of this kind, some challenges are anticipated with patient recruitment. The research team aims to mitigate this via appropriate counseling and use of our patient informed consent form, which is in a question-and-answer format.

The second challenge may be the accuracy of the retrospective data for the control group. This is an inherent issue with all retrospective studies. However, it is anticipated that key secondary outcomes will be accurately reported.

### Limitations

As with any study, there are limitations to acknowledge. First, cohort studies lack the same level of rigorous methodology as an RCT. However, based on the results of this pilot, we aim to proceed with a noninferiority RCT to compare this novel protocol to tNFET. Second, the retrospective nature of the control group may be associated with issues related to data entry.

### Implications

The implications of this study are that it may inform the future of FET. If primary outcomes are met such that the presence of the corpus luteum is maintained with P4mNFET, then a noninferiority RCT will be carried out to formally compare the two methods. Moving forward, this P4mNFET protocol may provide clinics the opportunity to perform FET with greater scheduling flexibility and comparable outcomes to established methods while potentially leading to cost-saving and increased efficiency.

## Appendix

Multimedia Appendix 1 Patient informed consent form for recruitment of patients to undergo the progesterone-modified natural cycle protocol for frozen embryo transfer at the Reproductive Services Unit of The Royal Women's Hospital, Melbourne.

Multimedia Appendix 2 Participant information sheet for use of progesterone pessary during progesterone-modified natural cycle protocol for frozen embryo transfer.

## Abbreviations

AE: adverse event
AFET: artificial cycle frozen embryo transfer
FET: frozen embryo transfer
hCG: human chorionic gonadotropin
LH: luteinizing hormone
LPS: luteal phase support
mNFET: modified natural cycle frozen embryo transfer
NFET: natural cycle frozen embryo transfer
P4: progesterone
P4mNFET: progesterone-modified natural cycle protocol for frozen embryo transfer
RCT: randomized controlled trial
RSU: Reproductive Services Unit
RWH: Royal Women’s Hospital
tNFET: true natural cycle frozen embryo transfer

## Data Availability

The datasets generated or analyzed during this study are available from the corresponding author upon reasonable request.
