# Supplementary material for: Feasibility of a Progesterone-Modified Natural Protocol for Frozen Embryo Transfer: Protocol for a Pilot Cohort Study
Source: JMIR Res Protoc. 2025 Apr 11;14:e66579. doi: 10.2196/66579 (PMC12032497; doi:10.2196/66579)
Supplement: Multimedia Appendix 1 [file resprot_v14i1e66579_app1.pdf]

Participant Information Sheet/Consent Form

Interventional Study - *Adult providing own consent*

The Royal Women's Hospital

**Title**	Feasibility of a progesterone modified natural cycle for frozen embryo transfer: a pilot cohort study
**Short Title**	Novel approach for frozen embryo transfer using progesterone
**Coordinating Principal Investigator/ Principal Investigator**	Dr Wan Tinn Teh<br>A/Prof Alex Polyakov<br>Dr Hector Georgiou
**Associate Investigator(s)**	Alexandra Churchill
**Location**	The Royal Women's Hospital

Part 1 What does my participation involve?

1 Introduction

You are invited to take part in this research project. This is because you are planning to use your frozen embryo to get pregnant, in what is medically called a Frozen Embryo Transfer (FET) cycle, and you have regular menstrual cycles. The aim of this project is to investigate a new approach in carrying out your frozen embryo transfer cycle.

This Participant Information Sheet/Consent Form (PICF) tells you about the research project. It explains the tests and treatments involved. Knowing what is involved will help you decide if you want to take part in the research.

Please read this information carefully. Ask questions about anything that you don't understand or want to know more about. Before deciding whether or not to take part, you might want to talk about it with a relative, friend or your local doctor.

Participation in this research is voluntary. If you don't wish to take part, you don't have to. You will receive the best possible care whether or not you take part.

If you decide you want to take part in the research project, you will be asked to sign the consent section. By signing it you are telling us that you:

• Understand what you have read
• Consent to take part in the research project
• Consent to have the tests and treatments that are described
• Consent to the use of your personal and health information as described.

You will be given a copy of this Participant Information and Consent Form to keep.

2 What is the purpose of this research?

When a woman has frozen embryos from a previous In Vitro Fertilisation (IVF) cycle, a treatment called a frozen embryo transfer, or FET cycle, is required in order to place an embryo back in the uterus (womb) at the right time – the so-called window of implantation. There are several accepted methods of carrying out a FET cycle, each with their own advantages and disadvantages. All types of FET cycles involve the administration of a hormone called progesterone, which, although naturally produced in the body, may be placed in the vagina in the form of a pessary, with the timing of commencing this pessary related to the specific FET cycle utilised.

A natural FET cycle, in which natural ovulation is relied on to prepare the lining of the uterus (known as the 'womb'), is increasingly accepted as one with the best protocols. However it may require multiple clinic visits for blood tests and scans, which can be inconvenient. In a natural FET, progesterone supplementation is started after a woman naturally ovulates.

The aim of this research is to investigate a variation in the timing of progesterone start on pregnancy outcomes, whereby it is commenced *before* ovulation. A similar study carried out abroad demonstrated similar outcomes in terms of pregnancy and live birth rates, with a possible reduction in clinic visits.

3 What does participation in this research involve?

You have been offered to be enrolled in this study because you meet the eligibility criteria and have indicated that you agree to be contacted for research purposes on your initial IVF consent form.

If you choose to participate, you will be required to sign the consent form.

Once you have provided your consent, you will be advised to call us with your next period. We will then schedule an internal (vaginal) ultrasound scan for you a few days after your period ends. This is the same as with any FET cycle. Based on the scan findings, the clinician scanning you may recommend a further scan or some blood tests. Once the ultrasound and blood test criteria for scheduling your embryo transfer are met, you will be advised to start the progesterone pessaries and will be informed of your embryo transfer day and time.

Your embryo transfer will take place as normal, with an internal examination and a transabdominal (via the tummy) ultrasound scan. The doctor scanning you will take an extra 1-2 minutes prior to your embryo transfer to scan your ovaries. This does not carry any risks to you or your embryo.

You will then be able to go home and we will advise you of the day of your pregnancy blood test. If the result is positive then we will arrange for you to have your first pregnancy scan a few weeks thereafter. If all is well on the scan then you will be able to transfer your care to an obstetrician. For the purposes of this study, we will contact (phone or email) you one more time at 12 weeks' of gestation to ensure that all is progressing well.

In the case of a negative result, you should discuss with your treating team regarding the next steps.

There are no additional costs associated with participating in this research project, nor will you be paid. As a gesture of gratitude for your participation in the study, you will receive a free supply of the progesterone pessaries required for your treatment.

Your participation in the study will end once we contact you at 12 weeks of gestation in the case of a positive result, or at pregnancy test in the case of a negative result.

Below is a schedule of clinic visits for a patient with a 28 day menstrual cycle undertaking a progesterone modified natural FET as compared to a natural FET:

FET Protocol	Days 11-14	Day 19	Day 29	Day 43	Day 84
Progesterone modified natural cycle FET	1-2 vaginal ultrasound examinations<br>1 set of blood tests	Embryo transfer	Pregnancy blood test	Pregnancy vaginal ultrasound	Pregnancy outcome measurement (telephone consultation)
Natural FET	1-2 vaginal ultrasound examinations<br>2-4 set of blood tests	Embryo transfer	Pregnancy blood test	Pregnancy vaginal ultrasound	

4 What do I have to do?

As a part of enrolment in the study, you will have some hormone blood tests. These are often, but not always, required even when not participating in the study. There are no other extra requirements of you if you choose to enrol in the study. There are also no restrictions for you if you choose to enrol.

5 Other relevant information about the research project

Our study aims to recruit 20 women who will all receive the same treatment. There will be no separate treatment arms.

6 Do I have to take part in this research project?

Participation in any research project is voluntary. If you do not wish to take part, you do not have to. If you decide to take part and later change your mind, you are free to withdraw from the project at any stage.

If you do decide to take part, you will be given this PICF to sign and you will be given a copy to keep.

Your decision whether to take part or not to take part, or to take part and then withdraw, will not affect your routine treatment, your relationship with those treating you or your relationship with the Public Fertility Services or the Royal Women's Hospital.

Once the consent form for participation is signed, you are free to withdraw your consent should you change your mind at any point.

7 What are the alternatives to participation?

You do not have to take part in this research project to receive treatment at this hospital. If you decide not to enrol, this decision will be respected. The treatment you receive from the hospital hereon will be the same as any other patient and you will not be disadvantaged.

8 What are the possible benefits of taking part?

We cannot guarantee that you will receive any benefits from this research; however, results from this work may help improve fertility outcomes in the future.

9 What are the possible risks and disadvantages of taking part?

There are currently no known risks or disadvantages in participating in this study. As this is a novel treatment, we cannot be sure whether this treatment will lead to pregnancy rates that are higher, lower or unchanged compared to other options. Irrespective of the timing of progesterone start, there are some rare side effects that women may experience, including headaches, skin irritation, nausea, vomiting or constipation or diarrhea.

If you become upset or distressed as a result of your participation in the research, the study doctor will be able to arrange for counselling or other appropriate support. Any counselling or support will be provided by qualified staff who are not members of the research project team. This counselling will be provided free of charge.

10 What if new information arises during this research project?

Sometimes during the course of a research project, new information becomes available about the treatment that is being studied. If this happens, your study doctor will tell you about it and discuss with you whether you want to continue in the research project. If you decide to withdraw, your study doctor will make arrangements for your regular health care to continue. If you decide to continue in the research project you will be asked to sign an updated consent form.

Also, on receiving new information, your study doctor might consider it to be in your best interests to withdraw you from the research project. If this happens, they will explain the reasons and arrange for your regular healthcare to continue.

11 Can I have other treatments during this research project?

It is important to tell your study doctor and the study staff about any treatments or medications you may be taking.

12 What if I withdraw from this research project?

If you decide to withdraw from the project, please notify a member of the research team before you withdraw. This notice will allow that person or the research supervisor to discuss any health risks or special requirements linked to withdrawing.

If you do withdraw your consent during the research project, which you are entitled to do if this is your wish, the study doctor and relevant study staff will not collect additional personal information from you, although personal information already collected will be retained to ensure that the results of the research project can be measured properly and to comply with law. Data collected up to the time you withdraw will not form part of the research project results.

13 Could this research project be stopped unexpectedly?

This research project may be stopped unexpectedly for a variety of reasons. These may include reasons such as:

- The treatment being shown not to be effective
- The treatment being shown to work and not need further testing

14 What happens when the research project ends?

The end of your participation in this study depends on the cycle outcome. If the cycle results in a pregnancy, we will continue to follow the pregnancy development until 12 weeks' gestation. If the cycle does not result in pregnancy, the participation ends at this point. In both cases, you will continue your follow up and treatment at the Public Fertility Services as usual.

The end of the study itself will involve collation of de-identified data and data analysis. We aim to present our findings at departmental meetings and disseminate them more widely at relevant conferences and medical journals. If you wish, you can be involved in the dissemination of results once the study has been analysed and reported.

Part 2 How is the research project being conducted?

15 What will happen to information about me?

By signing the consent form you consent to the study doctor and relevant research staff collecting and using personal information about you for the research project. Any information obtained in connection with this research project that can identify you will remain confidential.

Routine medical data will be kept on secure hospital electronic medical record file or on paper file in a secure locked room, which can be accessed securely by your treating team.

Any extra personal or health information obtained for the purpose of the study will be kept either on a secure server operated by the Royal Women's Hospital and paper files will be kept in a locked filing cabinet in a locked room within the hospital. The only people to have access to this data are:

- The research team involved in this project, who come from the Royal Women's Hospital
- The Royal Women's Hospital Research Office

Your information will only be used for the purpose of this research project and standard medical care, and it will only be disclosed with your permission, except as required by law.

Information about you may be obtained from your health records held at this and other health services for the purpose of this research. By signing the consent form you agree to the study team accessing health records if they are relevant to your participation in this research project.

It is anticipated that the results of this research project will be published and/or presented in a variety of forums. In any publication and/or presentation, information will be provided in such a way that you cannot be identified, as all data is analysed and presented anonymously.

Information about your participation in this research project may be recorded in your health records.

In accordance with relevant Australian and Victorian privacy and other relevant laws, you have the right to request access to your information collected and stored by the research team. You also have the right to request that any information with which you disagree be corrected. Please contact the study team member named at the end of this document if you would like to access your information.

Any information obtained for the purpose of this research project and for the future research that can identify you will be treated as confidential and securely stored. It will be disclosed only with your permission, or as required by law.

Please note, any direct or indirect profits made secondary to this project are not entitled to be shared with participants.

16 Complaints and compensation

If you suffer any injuries or complications as a result of this research project, you should contact the study team as soon as possible and you will be assisted with arranging appropriate medical treatment. If you are eligible for Medicare, you can receive any medical treatment required to treat the injury or complication, free of charge, as a public patient in any Australian public hospital.

The person you may need to contact will depend on the nature of your query. If you require further information concerning this project or if you have any medical problems which may be related to your involvement in the project (for example, any side effects), you can contact the principal study doctor on 8345 3200 or any of the following people:

<u>Clinical contact person</u>

Name: Dr Hector Georgiou
Position: Consultant Obstetrician & Gynaecologist, The Royal Women's Hospital
Telephone: (03) 8345 3200
Email: Ektoras.Georgiou@thewomens.org.au

For matters relating to research being conducted at the Women's, the details of the local site complaints person are:

<u>Complaints contact person</u>

Name: Consumer Liaison
Position: The Women's Consumer Liaison Team
Telephone: 8345 2290
Email: Consumer.liaison@thewomens.org.au

If you have any complaints about any aspect of the project, the way it is being conducted or any questions about being a research participant, then you may contact:

<u>Local Human Research Ethics Committee - Office contact (Research Governance Officer)</u>

Name: Research Office
Position: Women's Research Governance Officer
Telephone: 8345 3716
Email: Research.office@thewomens.org.au

Participation is voluntary. If you do not wish to take part you don't have to. If you decide to take part and later change your mind, you are able to withdraw at any stage.

17 Who is organising and funding the research?

This research project is being conducted by the Reproductive Services Unit at the Royal Women's Hospital. We have not received any specific funding for this project.

18 Who has reviewed the research project?

All research in Australia involving humans is reviewed by an independent group of people called a Human Research Ethics Committee (HREC). The ethical aspects of this research project have been approved by the HREC of the Royal Women's Hospital.

This project will be carried out according to the *National Statement on Ethical Conduct in Human Research (2007)*. This statement has been developed to protect the interests of people who agree to participate in human research studies.

Approval has been given by the Royal Women's Hospital to carry out the research and supervise the standard of care.

Consent Form - *Adult providing own consent*

**Title**	Feasibility of a progesterone modified natural cycle protocol for frozen embryo transfer: a pilot cohort study
**Short Title**	New approach for frozen embryo transfer
**Coordinating Principal Investigator/ Principal Investigator**	Dr Wan Tinn Teh<br>A/Prof Alex Polyakov<br>Dr Hector Georgiou
**Associate Investigator(s)**	Alexandra Churchill
**Location**	The Royal Women's Hospital

Consent Agreement

I have read the Participant Information Sheet or someone has read it to me in a language that I understand.

I understand the purposes, procedures and risks of the research described in the project.

I give permission for my doctors, other health professionals, hospitals or laboratories outside this hospital to release information to the Royal Women's Hospital concerning my treatment for the purposes of this project. I understand that such information will remain confidential.

I have had an opportunity to ask questions and I am satisfied with the answers I have received.

I freely agree to participate in this research project as described and understand that I am free to withdraw at any time during the study without affecting my future health care.

I understand that I will be given a signed copy of this document to keep.

I wish to receive a summary of the results at conclusion of the study ☐ Yes ☐ No

Please provide email address ………………………………………………………

Declaration by Participant – for participants who have read the information

Name of Participant (please print) ______________________________
Signature ______________________ Date ______________________

Declaration - for participants unable to read the information and consent form
Witness to the informed consent process<br>Name (please print) ______________________________
Signature ______________________ Date ______________________<br>* Witness is not to be the Investigator, a member of the study team or their delegate. Witness must be 18 years or older.

Declaration by Study Doctor/Senior Researcher†

I have given a verbal explanation of the research project, its procedures and risks and I believe that the participant has understood that explanation.

Name of Study Doctor/<br>Senior Researcher† (please print) ______________________________
Signature ______________________ Date ______________________

† A senior member of the research team must provide the explanation of, and information concerning, the research project.

Note: All parties signing the consent section must date their own signature.

I understand that, if I decide to discontinue the study treatment, I may be asked to attend follow-up visits to allow collection of information regarding my health status. Alternatively, a member of the research team may request my permission to obtain access to my medical records for collection of follow-up information for the purposes of research and analysis.

the women's
the royal women's hospital
victoria australia

Form for Withdrawal of Participation - *Adult providing own consent*

**Title**	Feasibility of a novel modified natural cycle with progesterone protocol for frozen embryo transfer: a pilot study
**Short Title**	New approach for frozen embryo transfer using progesterone
**Coordinating Principal Investigator/ Principal Investigator**	Dr Wan Tinn Teh<br>A/Prof Alex Polyakov<br>Dr Hector Georgiou
**Associate Investigator(s)**	Alexandra Churchill
**Location**	The Royal Women's Hospital

Declaration by Participant of a research project

I wish to withdraw from participation in the above research project and understand that such withdrawal will not affect my routine treatment, my relationship with those treating me or my relationship with the Royal Women's Hospital.

Name of Participant (please print) ______________________________

Signature ______________________ Date ______________________

Declaration by Study Doctor/Senior Researcher†

I have given a verbal explanation of the implications of withdrawal from the research project and I believe that the participant has understood that explanation.

Name of Study Doctor/
Senior Researcher† (please print) ______________________________

Signature ______________________ Date ______________________

† A senior member of the research team must provide the explanation of and information concerning withdrawal from the research project.

Note: All parties signing the consent section must date their own signature.
