# Supplementary material for: Feasibility of a Progesterone-Modified Natural Protocol for Frozen Embryo Transfer: Protocol for a Pilot Cohort Study
Source: JMIR Res Protoc. 2025 Apr 11;14:e66579. doi: 10.2196/66579 (PMC12032497; doi:10.2196/66579)
Supplement: Multimedia Appendix 2 [file resprot_v14i1e66579_app2.pdf]

Participant Information Sheet: Oripro Progesterone Pessary

Feasibility of a Progesterone Modified Natural Protocol for Frozen Embryo Transfer: A Pilot Cohort Study

the women's
the royal women's hospital
victoria australia

About the Oripro (200mg) Progesterone Pessary

By participating in this project, you are consenting to undergoing a relatively new variation in timing of when progesterone hormone is administered to you during your frozen embryo transfer – i.e., *before* you ovulate, as opposed to after.

Although naturally produced in your body, when given as an additional medication, such as in the form of a progesterone intra-vaginal pessary during fertility treatment, it can support your uterine lining to accept your frozen embryo, and help maintains pregnancy in the early stages.

You will be prescribed 2x pessaries to be inserted, 1x in the morning and 1x at night, from enrolment into the study until 8 weeks of pregnancy. Each pessary contains 200mg of progesterone hormone.

How to use the Oripro Progesterone Pessaries

- For a visual depiction and more information on how to use the Oripro progesterone pessaries, please see: https://vimeo.com/590889761/95a20febcd?share=copy
- Oripro must be inserted into the vagina, NOT taken orally or by any other method
- At time of use, and using clean, dry hands, remove 1 pessary from packaging, and insert it into your vaginal canal with either a lying or squatting position, as far as you are able to. You may choose to use Oripro's pessary applicator to assist with this
- Store the individual pessaries in their box, in a cool, dark, place in between doses

Side effects

- There are some *rare* side effects with progesterone supplementation, not unique to our approach, including:
    - *Headache, mood changes*
    - *Skin changes or irritation*
    - *Diarrhea, constipation, nausea, vomiting*
- It is important that you contact us with any side effects that you may be experiencing during your enrolment in the study. Kindly email these to us at rsu.research@thewomens.org.au
- Long-term side effects are not expected as part of treatment. If concerned about your symptoms, please reach out

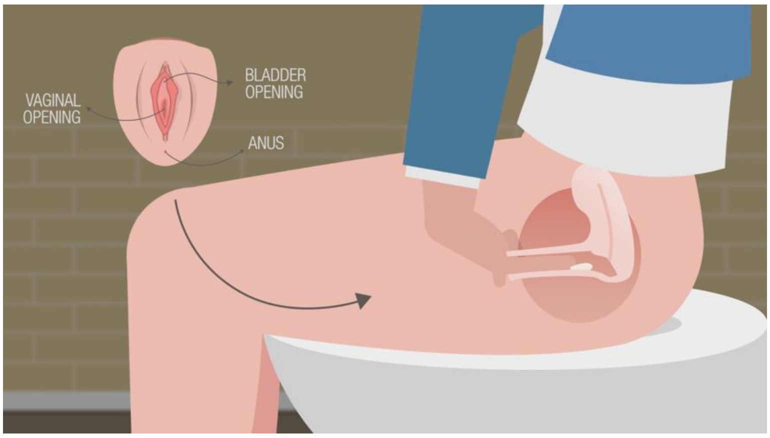

Source: https://vimeo.com/590889761/95a20febcd?share=copy

When can I NOT use progesterone pessaries?

Certain health conditions put women at risk of harmful complications when using any form of progesterone supplementation. If any of these apply to you, please contact us as you may not be eligible for this research project: rsu.research@thewomens.org.au

- *Abnormal vaginal bleeding, liver disease, clotting disorders, certain cancers (past or current), ectopic pregnancy or missed abortion*
- *Any other health conditions that you are concerned about*
